# Surface Acoustic Wave Sensors for Wireless Temperature Measurements above 1200 Degree Celsius

**DOI:** 10.3390/s24154945

**Published:** 2024-07-30

**Authors:** Hong Zhang, Danyu Mu, Zichao Zhang, Jikai Zhang, Jiabao Sun, Hao Jin

**Affiliations:** 1 College of Information Science & Electronic Engineering, Zhejiang University, Hangzhou 310027, China; hong.zhang@zju.edu.cn (H.Z.); 12231023@zju.edu.cn (D.M.); zhjk19980614@163.com (J.Z.); 2 Innovation and Research Institute of HIWING Technology Academy, Beijing 100074, China; zzc_asic@163.com; 3 Micro-Nano Fabrication Center, Zhejiang University, Hangzhou 310027, China; jbsun@zju.edu.cn; 4 International Campus, Zhejiang University, Haining 314400, China

**Keywords:** high-temperature stability, wireless sensing, surface acoustic wave, quasi-shear wave, lanthanum gallium silicate, multilayer composite electrode

## Abstract

High-temperature wireless sensing is crucial for monitoring combustion chambers and turbine stators in aeroengines, where surface temperatures can reach up to 1200 °C. Surface Acoustic Wave (SAW) temperature sensors are an excellent choice for these measurements. However, at extreme temperatures, they face issues such as agglomeration and recrystallization of electrodes, leading to loss of conductivity and reduced quality factor, hindering effective wireless signal transmission. This study develops an LGS SAW sensor with a Pt-10%Rh/Zr/Pt-10%Rh/Zr/Pt-10%Rh/Zr multilayer composite electrode structure to address these challenges. We demonstrate that the sensor can achieve wireless temperature measurements from room temperature to 1200 °C with an accuracy of 1.59%. The composite electrodes excite a quasi-shear wave on the LGS substrate, maintaining a Q-factor of 3526 at room temperature, providing an initial assurance for the strength of the wireless interrogation echo signal. The sensor operates stably for 2.18 h at 1200 °C before adhesion loss between the composite electrode and the substrate causes a sudden increase in resonant frequency. This study highlights the durability of the proposed electrode materials and structure at extreme temperatures and suggests future research to improve adhesion and extend the sensor’s lifespan, thereby enhancing the reliability and effectiveness of high-temperature wireless sensing in aerospace applications.

## 1. Introduction

As the core component of an aircraft, an aeroengine is a complex and highly precise thermomechanical system that operates under extreme high temperatures for prolonged periods [1,2]. Accurate measurement of transient temperatures is crucial in numerous locations within the engine, such as the combustion chamber and turbine stators. The walls of the combustion chamber and turbine stators are directly exposed to the combustion process and high-temperature gases, placing them at the forefront of critical thermal load areas. Surface temperatures in these areas can reach up to 1200 °C [3]. Monitoring the temperature of these regions is essential for ensuring the engine’s safe operation, maintaining high-efficiency performance, and extending its service life. Accurate temperature data can mitigate potential failures, reduce maintenance costs, and enhance overall performance.

These hot-end components are located in confined working environments with complex part structures, which limit the use of conventional and battery-powered temperature sensors. Surface Acoustic Wave (SAW) wireless passive temperature measurement technology offers a promising solution for this field of temperature measurement. This technology is highly beneficial in extreme environments due to its robustness, lack of need for external power sources, and ability to transmit data over distances without physical connections. Consequently, SAW wireless sensing systems can minimize the footprint of structural health monitoring systems, avoid the costs of expensive wiring and safety verification, and improve system operational stability [4,5]. The wireless nature of these sensors allows for real-time monitoring in harsh environments, providing critical data without the need for intrusive installations. This is particularly advantageous in aerospace applications where space and weight are at a premium.

The initial ideas for using SAW sensors as temperature sensors were proposed around 40 years ago [6,7] and have been continuously developed since then. In 2005, M. N. Hamidon et al. fabricated high-temperature SAW sensors based on gallium orthophosphate, using a lift-off technique, and tested them for high-frequency applications at temperatures up to 600 °C [8]. The direct deposition of Pt onto lanthanum gallium silicate (LGS) at a low deposition rate (5 nm/min) and at a high temperature (630 °C) allowed for high-crystalline-quality Pt films showing an epitaxial relationship with the substrate and a mean grain size close to 340 nm, and LGS SAW sensors based on this film could work at 800 °C for at least 3 h [9]. Q. Shan et al. proposed the fabrication processes of the grooved LGS SAW sensors and showed the sensors could work at 1000 °C for 12 h [10]. In the same year, J. Luo et al. proposed a LGS SAW sensor based on a Pt-Al_2_O_3_ composite electrode (with Pt sandwiched between two layers of Al_2_O_3_) and achieved a temperature measurement range of 80–1000 °C [11]. Self-sustained, high-temperature stable SAW sensors based on calcium tantalum gallium silicate (GTGS) with Al-Ru electrodes demonstrated high-temperature stability up to at least 700 °C [12].

However, the aforementioned SAW sensors’ high performance for high-temperature measurement is mainly based on wired test results. There are very few evaluations of SAW sensors’ performance based on wireless testing, especially at high temperatures. At elevated temperatures, electrodes suffer from issues such as agglomeration, recrystallization, and oxidation, which gradually cause a loss of conductivity. This leads to a sharp decline in the device’s quality factor (Q-factor) [9], preventing the generation of effective echo signals for the reader to detect and identify, thus impeding the implementation of wireless temperature measurement. In the existing literature, B. François et al. used LGS SAW resonators for wireless temperature measurements up to 700 °C for durations ranging from 10 to 40 h [13]. In the same year, P. Zhang et al. reported an LGS SAW sensor that achieved wired temperature sensing at temperatures up to 900 °C and wireless temperature sensing up to 700 °C [14]. A passive wireless SAW sensor system for detecting temperature variations of concrete from −10 °C to 100 °C is described, where the sensors were embedded within a 2-inch thick concrete cover [15]. B. François et al. proposed a high-temperature packaging method for SAW sensors acting as passive wireless sensors, and the reliability of this process applied to differential SAW sensors is demonstrated by the wireless interrogation of a quartz-based SAW differential sensor from room temperature to 480 °C [16]. Zhen Wu et al. proposed a SAW temperature monitoring system for gas-insulated switchgears with a temperature measurement range of 0 °C to 100 °C [17]. To our knowledge, there are currently no reports in the literature concerning the wireless test results of LGS SAW sensors above 1200 °C. This gap in the literature underscores the need for further research and development in wireless, high-temperature SAW sensor technology.

LGS is a promising piezoelectric material for high-temperature sensing due to its lack of phase transitions up to its melting point (approximately 1450 °C) [18,19,20,21,22]. The key to the high-temperature stability of the LGS SAW sensor lies in the survival of the metal electrodes in high-temperature environments, as conventional metal electrodes are prone to agglomeration and recrystallization at high temperatures, affecting sensor performance [11,23,24,25]. We proposed a novel composite electrode structure that effectively prevents electrode agglomeration and recrystallization at high temperatures. Compared to other electrode types, this composite electrode structure, composed of Pt-10%Rh/Zr/Pt-10%Rh/Zr/Pt-10%Rh/Zr (150/30/150/30/150/10 nm) layers, offers higher thermal stability. The LGS SAW sensor based on this composite electrode can excite a quasi-shear wave mode distinct from conventional Rayleigh wave modes. This mode has a higher resonant frequency than conventional Rayleigh wave mode, facilitating the miniaturization of the sensing antenna and possessing a high Q-factor. This enables wireless temperature measurement over a wide range up to 1200 °C, with a full-scale measurement accuracy of 1.59%. Experimental results demonstrate that this sensor is capable of monitoring the temperature of the combustion chamber walls and turbine stators in aircraft engines in real-time. This innovation represents a significant advancement in the field of high-temperature wireless sensing, offering reliable and accurate temperature monitoring in the most demanding conditions.

## 2. Design and Tests of High-Temperature Sensors

### 2.1. Modelling and Simulations

**Figure 1:**
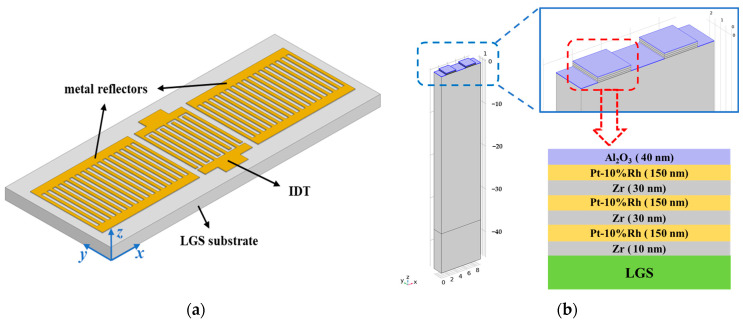
Structural schematic and simulation model of the LGS SAW Sensor: (**a**) The structural schematic of the LGS SAW sensor, showing one interdigital transducer (IDT) and two metal reflectors; (**b**) The COMSOL simulation model of a pair of crossing fingers with a metallization ratio of 0.5. The inset details the multilayer thin-film composite electrode structure.

The structural schematic of the LGS SAW sensor adopted in this study is shown in Figure 1a. It consists of one interdigital transducer (IDT) and two metal reflector gratings located on the LGS substrate, presenting a periodic structure. Therefore, to reduce the computational load of the simulation model, a pair of crossing fingers with a metallization ratio of 0.5 was chosen to simulate the SAW temperature sensor in the COMSOL Multiphysics software (version 6.2), as shown in Figure 1b. The substrate material was LGS, and a rotating coordinate system was utilized, setting the Euler angles of the crystal to (0°, 138.5°, 26.7°), where the LGS SAW has a smaller angle of acoustic wave diffraction and hence achieves a relatively high Q-factor. To endure high temperatures up to 1200 °C, a multilayer thin-film composite electrode structure was utilized in the simulation. The composite electrode structure was Pt-10%Rh/Zr/Pt-10%Rh/Zr/Pt-10%Rh/Zr (150/30/150/30/150/10 nm), as shown in the inset of Figure 1b. The simulated width of the cross-finger was 2.3 microns, and the wavelength (λ) was 9.2 microns. The model’s substrate thickness was 46 microns, with a Perfectly Matched Layer (PML) added to the bottom of the model to absorb acoustic waves propagating towards the bottom of the material, preventing reflections of acoustic waves from interfering with the modal simulation. The width and depth of the model’s substrate were 9.2 and 2.3 microns, respectively. In the solid mechanics interface, the boundary conditions on the upper and lower surfaces were set as free and fixed, respectively, while two sets of periodic boundary conditions were applied to the model: one set to the front and back, and another set to the left and right sides. In the electrostatics interface, one electrode was grounded, while the other was connected to a 1-volt voltage source. Two sets of periodic boundary conditions are applied to the non-metallic region of the model: one set to the front and back, and another set to the left and right sides. The governing equations for the piezoelectric analysis in COMSOL Multiphysics are as follows [26]:

(1) $$\left[\nabla_{s}\right]\left(\left[c\right]{\left[\nabla_{s}\right]}^{T}\left\{u\right\}+{\left[e\right]}^{T}{\left[\nabla \right]}^{T}\left\{\phi \right\}\right)-\rho \frac{\partial^{2}}{\partial t^{2}}\left\{u\right\}=0,$$

(2) $$\left[\nabla \right]\left(\left[e\right]{\left[\nabla_{s}\right]}^{T}\left\{u\right\}-\left[\epsilon \right]{\left[\nabla \right]}^{T}\left\{\phi \right\}\right)=0,$$

where $\left[c\right]$ is the stiffness matrix, $\left[e\right]$ is piezoelectric the stress matrix, $\left\{u\right\}$ is the displacement matrix, $\left[\epsilon \right]$ is the permittivity matrix, and $\left\{\phi \right\}$ is the electrical potential of the piezoelectric materials.

**Figure 2:**
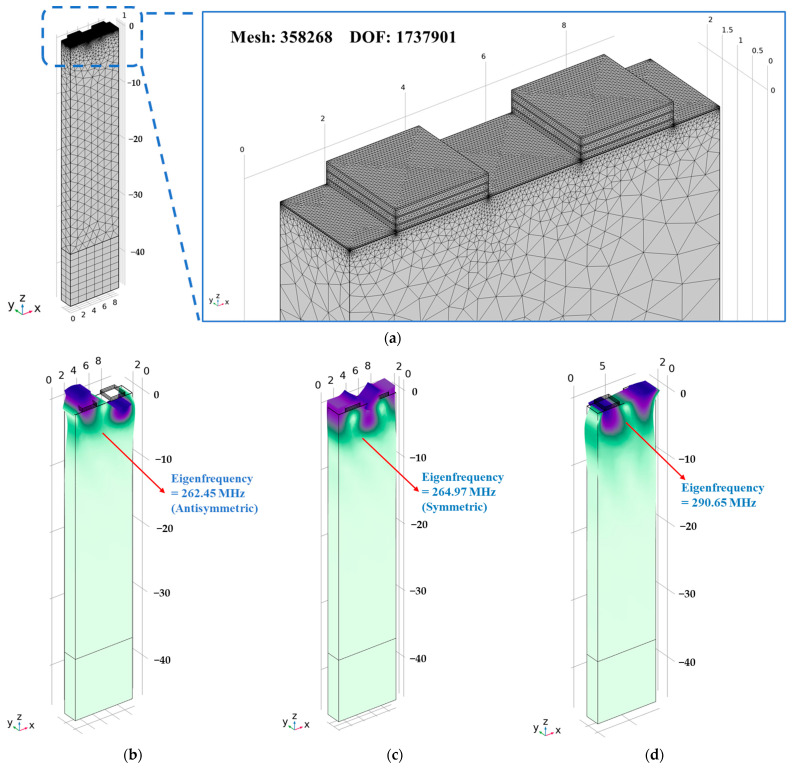
Finite element model mesh and the corresponding eigenfrequency study results: (**a**) Mesh division of the simulation model, with a total of 358,268 elements and 1,737,901 degrees of freedom (DOF); (**b**) Antisymmetric and (**c**) symmetric Rayleigh wave modes; (**d**) Quasi-shear wave mode.

**Figure 3:**
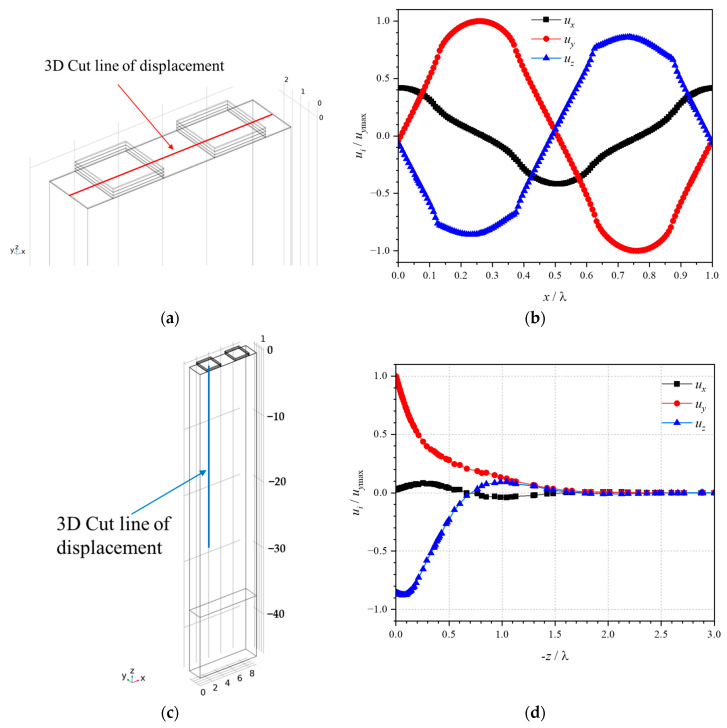
Distribution diagram of normalized particle displacement components (*u_i_*/*u_y_*_max_, *i* = *x*, *y*, *z*) for the quasi-shear wave mode along three orthogonal directions on the LGS surface and into the interior of the LGS: (**a**) Simulation calculation area of displacement on the surface highlighted by a red 3D cut line and (**b**) the corresponding particle displacement calculation results of the quasi-shear wave; (**c**) Simulation calculation area of displacement into the interior of the LGS from the surface highlighted by a blue 3D cut line and (**d**) the corresponding particle displacement calculation results of the quasi-shear wave mode.

An eigenfrequency study was used to compute the eigenmodes and eigenfrequencies of the LGS SAW sensor, and the simulation results are shown in Figure 2. Figure 2a shows the mesh division and degrees of freedom during the eigenfrequency study of the model. The element size is set to extra fine, resulting in 358,268 elements and 1,737,901 degrees of freedom. The LGS SAW sensor with such a composite thin-film electrode structure can not only excite a conventional Rayleigh wave mode, which has both antisymmetric and symmetric modes [27], but also excite a quasi-shear wave mode, which has a higher acoustic velocity than Rayleigh wave mode and is derived from bulk acoustic waves confined by such thick electrodes [28,29]. The antisymmetric and symmetric Rayleigh wave modes are shown in Figure 2b and Figure 2c, respectively, and the quasi-shear wave mode is shown in Figure 2d. The eigenfrequencies of the antisymmetric and symmetric Rayleigh wave modes are 262.45 MHz and 264.97 MHz, respectively, as shown in Figure 2b,c. The eigenfrequency of the quasi-shear wave mode is 290.65 MHz, as shown in Figure 2d. The eigenfrequency of quasi-shear wave mode is over 25 MHz higher than that of Rayleigh wave mode. The higher eigenfrequency facilitates the miniaturization of the sensing antenna connected to the LGS SAW sensor, as well as the absorption and emission of electromagnetic waves by the entire wireless sensing unit in confined installation spaces. The distribution diagram of the normalized particle displacement components (*u_i_*/*u_y_*_max_, *i* = *x*, *y*, *z*) of the quasi-shear wave mode along three orthogonal directions on the LGS surface and into the interior of the LGS was also simulated, as shown in Figure 3. Figure 3a shows the simulation calculation area of displacement on the surface highlighted by a red three-dimensional (3D) cut line, and the calculation results are presented in Figure 3b, where the quasi-shear wave mode has the maximum displacement component in the y-direction and the minimum one in the x-direction (the SAW propagation direction). This indicates that the quasi-shear wave primarily consists of horizontal and vertical shear wave components, with only a small number of longitudinal wave components. Note that the number of data points in Figure 3b depends on the element size along the red 3D cut line. Figure 3c shows the simulation calculation area of displacement into the interior of the LGS from the surface highlighted by a blue 3D cut line, and the calculation results are presented in Figure 3d, where the amplitudes of the displacement components decay to nearly zero at the depth (represented by -z) of 2λ. This indicates that the vibrational energy of the quasi-shear wave is confined to the surface of the LGS substrate, classifying it as a surface acoustic wave.

The eigenfrequency study of the model indicates that the quasi-shear wave has a higher eigenfrequency compared to conventional Rayleigh waves, which is advantageous for the miniaturization of sensing antennas. Additionally, there are no other resonant modes within tens of MHz of its eigenfrequency, whereas the symmetric and antisymmetric modes of Rayleigh waves are only 2.52 MHz apart. Therefore, using quasi-shear waves for wireless temperature sensing is likely to be more beneficial for maintaining the purity of the echo signal and improving the accuracy of wireless temperature measurements.

### 2.2. Sensor Fabrication

**Figure 4:**
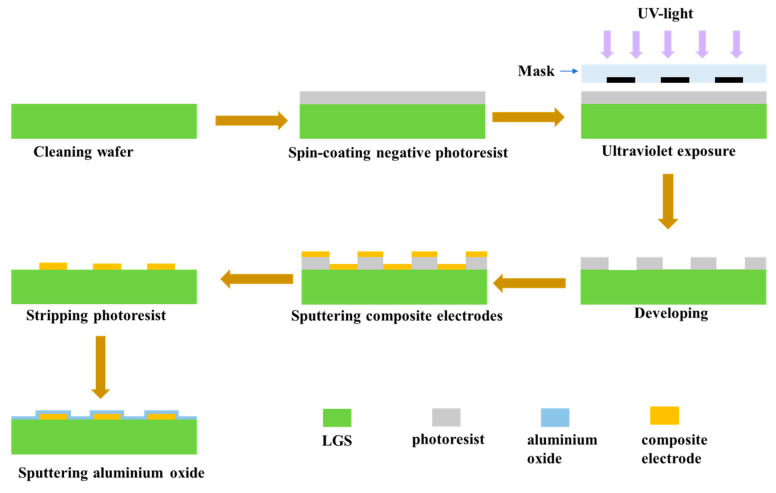
Schematic of the main fabrication process of LGS SAW sensors.

**Figure 5:**
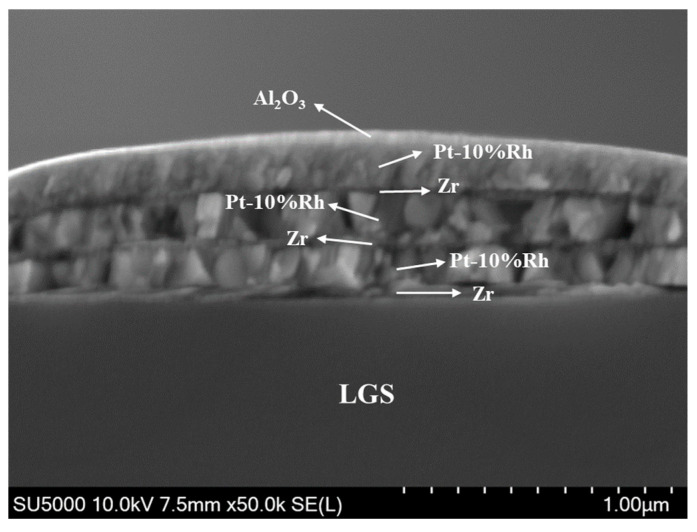
Scanning electron microscopy (SEM) image of the multilayer composite electrode structure.

The preparation of the high-temperature LGS SAW sensor involved several steps, as shown in Figure 4. The thickness of the 4-inch LGS wafer (Fomos-Materials Co. Ltd., Moscow, Russia) used to fabricate the sensors is 500 μm. The wafer is single-side polished, with the polished surface having a surface roughness (Ra) of ≤0.7 nm. The wafer was first prepared using commonly used, clean recipes that do not contain any acids. After cleaning, the wafer was coated with a negative photoresist, followed by ultraviolet exposure to transfer the pattern from the mask to the wafer after development. The multilayer thin-film electrode structure was then fabricated by magnetron sputtering (Discovery-635, Denton Vacuum Co. Ltd., Moorestown, NJ, USA) and stripping the photoresist. The electrode structure consists of Pt-10%Rh/Zr/Pt-10%Rh/Zr/Pt-10%Rh/Zr (150/30/150/30/150/10 nm), where a 10 nm Zr was used as an adhesion layer. Figure 5 shows the scanning electron microscopy (SEM) image of the multilayer composite electrode structure. After fabricating the multilayer thin-film composite electrode structure, a 40 nm alumina passivation layer was sputtered on the surface of the entire wafer to further enhance the temperature resistance of the sensor and provide extra mechanical protection. To improve electrode performance, high-temperature, high-vacuum pre-annealing was performed on the LGS SAW sensors to anneal defects in the Pt-10%Rh layers [25]. The sensors on the 4-inch LGS wafer were heated to 1000 °C in a 1 Pa base pressure infrared annealing furnace for one hour and then cooled to room temperature in a vacuum before removal from the chamber. Finally, the 4-inch LGS wafer was cut into discrete LGS SAW sensors for wireless temperature measurement.

**Figure 6:**
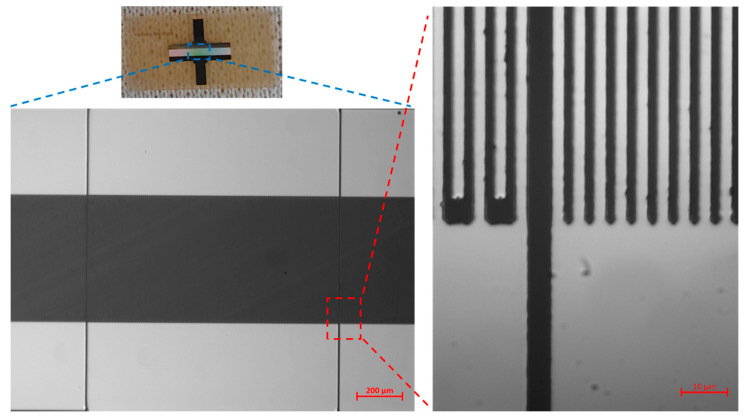
Photograph of the fabricated high-temperature LGS SAW sensor and magnified images of interdigital electrodes observed under a metallographic microscope.

A photograph of the prepared LGS SAW sensor and a magnified image of the interdigital electrodes taken under a metallographic microscope are shown in Figure 6. The morphology of the interdigital electrodes is intact and free of shorts and breaks. The LGS SAW sensor consists of one IDT and two metal reflector gratings. The IDT contains 120 pairs of electrodes with an electrode width of 2.3 microns and an acoustic aperture of 60 λ. Each metal reflector grating consists of 200 pairs of short-circuit bars, and the distance between the IDT and the adjacent metal reflector gratings is 0.625 λ.

### 2.3. Test System

**Figure 7:**
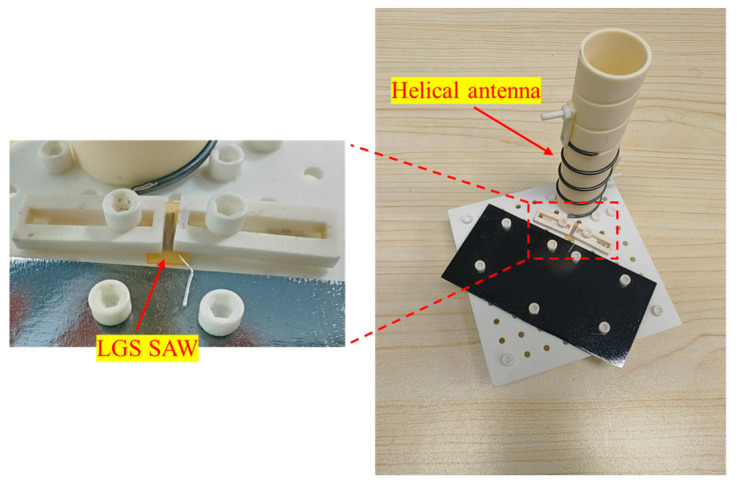
Integrated wireless sensing unit of the normal-mode helical antenna and the LGS SAW sensor.

The scattering parameters of the LGS SAW sensors were measured by a network analyzer (E5071C, Keysight, Santa Rosa, CA, USA). Based on the measured S-parameters, the LGS SAW sensor with excellent performance was selected to fabricate an integrated sensing unit for wireless temperature measurement. Figure 7 shows the integrated wireless sensing unit, which primarily consists of an LGS SAW sensor and a normal-mode helical antenna, supported by a ceramic fixture. The normal-mode helical antenna was chosen as the sensing antenna for the LGS sensor because it is air-loaded, allowing for a large antenna bandwidth and minimal sensitivity to temperature changes [30]. The LGS SAW sensor and the normal-mode helical antenna were electrically connected through a platinum-rhodium wire and shared a sheet of metal as the common ground plane. To save on testing costs, both the normal-mode helical antenna and the metal sheet were made of nickel and could be replaced if heavily oxidized after multiple cycles of wireless high-temperature testing.

**Figure 8:**
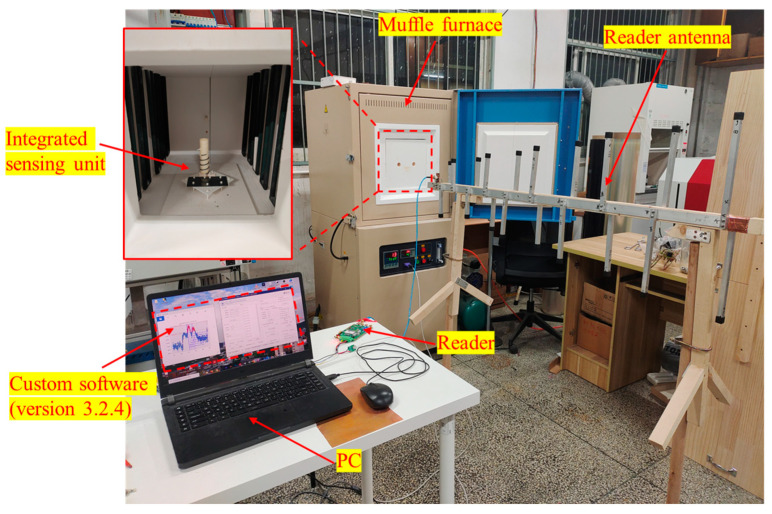
Photograph of the constructed wireless temperature sensing test system, including an integrated wireless sensing unit, a muffle furnace, a reader antenna, a reader, and custom software (version 3.2.4) installed on a personal computer terminal to control the operation of the reader.

The wireless temperature test platform is shown in Figure 8. The entire measurement system includes the aforementioned integrated wireless sensing unit, a muffle furnace, a reader antenna, a reader, and custom software (version 3.2.4) installed on a personal computer terminal to control the operation of the reader. The muffle furnace featured a 30-segment intelligent temperature controller to set the temperature as well as the heating and cooling times, utilizing a proportional integral derivative (PID) control system with a temperature control accuracy of ±1 °C. The furnace chamber was made of high-purity alumina, providing excellent insulation and thermal retention. To allow the interrogation of radio frequency signals to enter and exit the muffle furnace, enabling signal interaction between the integrated sensing unit and the reader antenna, the door of the muffle furnace was made of alumina ceramic fibers. To achieve a wide-range wireless temperature measurement up to 1200 °C, a log periodic dipole antenna (LPDA) was employed as the reader antenna, which is characterized by its wide frequency band, high gain, and good directivity. To reduce the quality requirements for the echo signal returned by the sensing unit, the reader architecture implemented a frequency sweep and power detection scheme in the frequency domain. The custom software to control the operation of the reader was written in Python 3.8.10.

Initially, this integrated wireless sensing unit was placed in a muffle furnace for heating to perform wireless temperature sensing calibration. Subsequently, wireless temperature sensing tests were conducted, and measurements of temperatures were derived from the previously fitted model and measured resonant frequencies. These tests aimed to validate the performance and reliability of the LGS SAW sensors in real-world high-temperature conditions, providing critical data that could be used to optimize sensor design and application.

## 3. Results and Discussion

### 3.1. Electrical Characteristics

**Figure 9:**
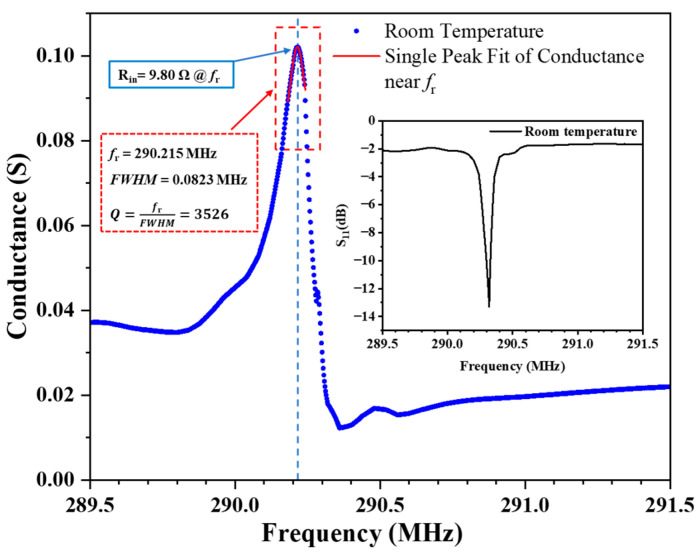
Conductance curve of the LGS SAW sensor calculated from the S-parameter curve measured at room temperature, as shown in the inset.

The S-parameter curve measured at room temperature (15 °C) is shown in the inset of Figure 9, with the minimum S_11_ value of −13.3 dB occurring at a frequency of 290.32 MHz. From the measured S-parameter curve, the conductance curve of the LGS SAW sensor can be calculated, as shown in Figure 9. The frequency at which the conductance curve reaches its maximum value is the resonant frequency ($f_{r}$) of the LGS SAW sensor, which is 290.215 MHz. This frequency shows a slight discrepancy from the eigenfrequency of the quasi-shear wave obtained in the simulation model in Section 2.1. This discrepancy mainly arises from the fabrication process errors of the sensor, the simplifications made in the simulation model compared to the actual device, and the differences between the material parameters used in the simulation and those of the materials actually used in sensor fabrication. Taking the reciprocal of the conductance curve reveals that the input impedance of the LGS SAW sensor at the resonant frequency is 9.80 ohms, which facilitates impedance matching for the normal-mode helical antenna. This is important because the input impedance of the normal-mode helical antenna is always small due to its physical dimensions and the way it radiates. The Q-factor of the LGS SAW sensor can also be derived from the conductance curve by dividing the resonant frequency by the full width at half maxi-mum (FWHM) of the resonance peak [31]. To accurately calculate the FWHM of the resonance peak and eliminate the influence of the secondary peak on the left side of the resonance peak, single peak fitting was performed using a Lorentzian function, which is widely used for single peak fitting in physical resonance phenomena. The data points selected for fitting are located within the red-dashed rectangular box in Figure 9. By dividing the resonant frequency by the FWHM obtained from the fitting, the Q-factor at the resonance point is determined to be approximately 3526. The high Q-factor at room temperature is a fundamental assurance for performing wireless wide-range temperature measurements (from room temperature to 1200 °C).

### 3.2. Wireless Temperature Measurement Range and Accuracy

**Figure 10:**
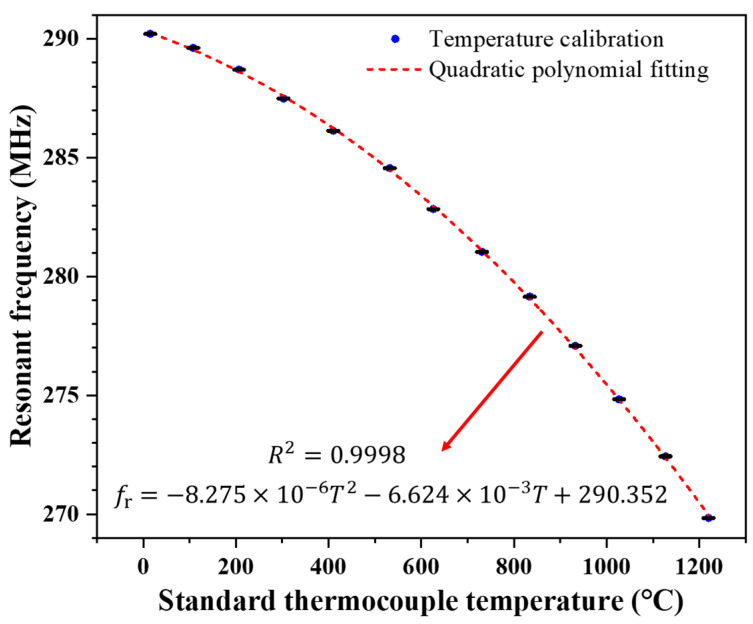
Frequency-temperature calibration curve of the LGS SAW sensor for wireless temperature measurement.

Before conducting wireless temperature measurements, the LGS sensor needs to be calibrated. The calibrated temperature range should exceed the actual temperature measurement range. In this study, the calibrated temperature range spanned from room temperature to 1220 °C, with room temperature being 15 °C. The calibration results are shown in Figure 10. The resonant frequency of the LGS SAW sensor at each temperature point in the calibration curve was measured five times, and the average value of these five measurements was taken as the resonant frequency for each calibration point. Note that the lengths of the error bars in Figure 10 are very short because we conducted the five repeated measurements at each calibration point only after the temperature inside the muffle furnace reached the set value and remained stable for 20 min. This ensured that the five resonant frequency readings at each calibration point were very close to each other, resulting in very short error bars in Figure 10. The resonance frequency changes nonlinearly as the temperature increases, and the calibration curves can be well-fitted by a quadratic polynomial, with the fitting formula as follows:

(3) $$f_{r}\left(T\right)=-8.275\times 10^{-6}T^{2}-6.624\times 10^{-3}T+290.352,$$

where $f_{r}\left(T\right)$ is the resonant frequency of the LGS SAW sensor at temperature T. The R-Square of the fitting model is 0.9998, indicating that the fitting model performs well and nearly all the variability of the measured data is around its mean.

**Figure 11:**
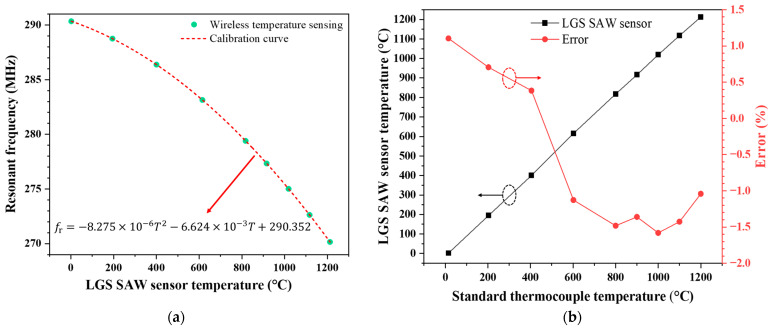
Wireless temperature sensing test results: (**a**) Resonant frequencies read by the reader and the corresponding temperatures of the LGS SAW sensor (calculated based on the calibration curve using the resonant frequencies); (**b**) Temperature measurement error of the LGS SAW temperature sensor compared to the standard thermocouple, where the error is defined as the difference between the standard thermocouple temperature and the LGS SAW sensor temperature divided by the full scale of temperature measurement (1200 °C).

After completing the wireless calibration of the LGS SAW temperature sensor, the wireless temperature sensing test was conducted, with the measurement range falling within the calibrated range. At each measurement temperature point, we ensured the temperature in the muffle furnace was stable for 20 min before taking the measurements, with the temperature measurement range spanning from room temperature to 1200 °C. The wireless temperature sensing test results are shown in Figure 11. The calibration curve in Figure 11a is the same as the one obtained in Figure 10. It can be seen that the temperatures measured by the LGS SAW sensor and their corresponding resonant frequencies fall precisely on the calibration curve in Figure 11a, confirming that the calibration curve used in this wireless temperature measurement is the calibration curve in Figure 11a. Figure 11b illustrates the temperature measurement errors calculated based on the temperatures measured by the standard thermocouple and the LGS SAW sensor, which indicates the high accuracy of the previously obtained calibration model and the high-temperature stability of the LGS SAW sensor. The formula for calculating the temperature measurement error ($\varepsilon_{r}$) is as follows:

(4) $$\varepsilon_{r}=\frac{T_{thermocuple}-T_{sensor}}{T_{FS}}\times 100\%,$$

where $T_{thermocuple}$ is the temperature measured by the standard thermocouple, $T_{sensor}$ is the temperature measured by the LGS SAW sensor, and $T_{FS}$ is the full-scale temperature range, which in this case is 1200 °C. The wireless temperature test results reveal that the sensor has a measurement range from room temperature to 1200 °C, with an accuracy within 1.59%FS.

**Figure 12:**
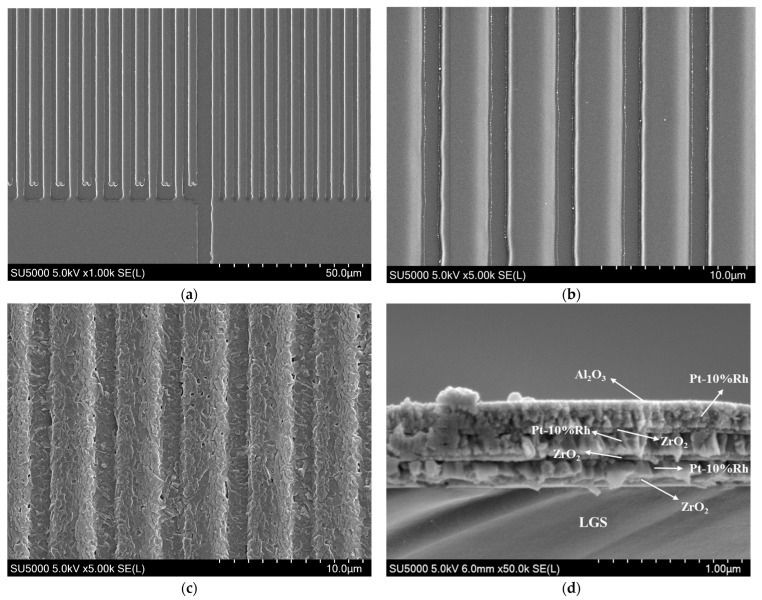
SEM images of Pt-10%Rh/Zr/Pt-10%Rh/Zr/Pt-10%Rh/Zr electrodes on LGS SAW sensors: (**a**,**b**) Surface topography of LGS SAW sensors before the full-range wireless temperature measurement evaluation; (**c**) Surface topography and (**d**) cross-sectional morphology of LGS sensors after the full-range wireless temperature measurement evaluation.

The surface morphologies of the LGS SAW sensors were characterized using a SEM before and after wireless temperature measurement over the full range, as shown in Figure 12. Before high-temperature measurement, the surface of the sensor is smooth, and there are no obvious particles, as seen in Figure 12a,b. Note that the metallization rate in Figure 12a,b, appears higher than that in Figure 6. This discrepancy is mainly due to two factors: first, fabrication process differences caused by contact lithography and immersion development; and second, the depth of field of the metallurgical microscope at 1000× magnification is much smaller than that of the scanning electron microscope. Therefore, when the metallurgical microscope is focused on the surface of the electrode center, regions with thinner electrode edges due to the wide angular distribution of magnetron sputtering cannot be resolved by the metallurgical microscope. After the full-range wireless temperature measurement evaluation, the electrode morphology remains intact, with no significant agglomeration observed in Figure 12c. The electrode surface is only slightly covered with particles, and no obvious recrystallization phenomena are present. In addition, Figure 12d shows that the adhesion of the composite electrode film to the LGS substrate is still very good after a high-temperature test. Combining Figure 12c,d, it can be concluded that the LGS SAW sensor remains in good condition and can continue to operate normally for an extended period.

The wide temperature measurement range and high accuracy reflect the excellent high-temperature stability of the LGS SAW sensors, which originates from the novel composite electrode structure (Pt-10%Rh/Zr/Pt-10%Rh/Zr/Pt-10%Rh/Zr) employed in the LGS SAW sensors combined with a 40 nm Al_2_O_3_ passivation layer. Firstly, the thick composite electrode excites a quasi-shear wave mode distinct from the conventional Rayleigh wave, with a Q-factor of 3526 at room temperature, as previously mentioned. This ensures the strength and signal-to-noise ratio of the wireless sensing signal. Secondly, the high-temperature endurance of the multilayer composite electrode is attributed to the 30 nm zirconium interlayers, which oxidize at high temperatures to form zirconia pinning layers. These layers restrict the atomic agglomeration and recrystallization of the platinum-rhodium alloy [32]. Additionally, extra energy is required to break the Al_2_O_3_ passivation layer before aggregation [12]. These synergistic effects ensure the morphological integrity of the electrode at high temperatures and enable these electrode materials to have high enough conductivity to make the LGS SAW sensor stably operate up to 1200 °C.

### 3.3. Lifespan and Failure Analysis

**Figure 13:**
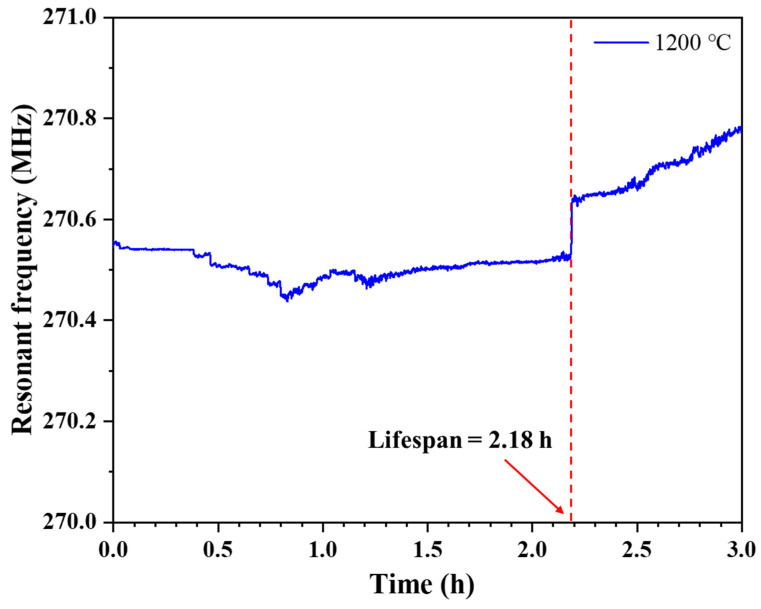
Resonant frequency of the LGS SAW sensor plotted against time during a 3-h period at 1200 °C.

To evaluate the high-temperature endurance of the LGS SAW sensor at 1200 °C, the calibrated temperature sensor was placed in a muffle furnace for continuous wire-less temperature measurement at 1200 °C for 3 h. The test results are shown in Figure 13. The LGS SAW sensor consistently maintained stable performance for 2.18 h, after which the resonant frequency experienced a sudden increase. This suggests that the operational lifespan of the LGS SAW sensor at the extreme temperature of 1200 °C is 2.18 h. However, since the sensor is unlikely to operate continuously at this extreme temperature, its normal operational lifespan is expected to be significantly longer than 2.18 h.

**Figure 14:**
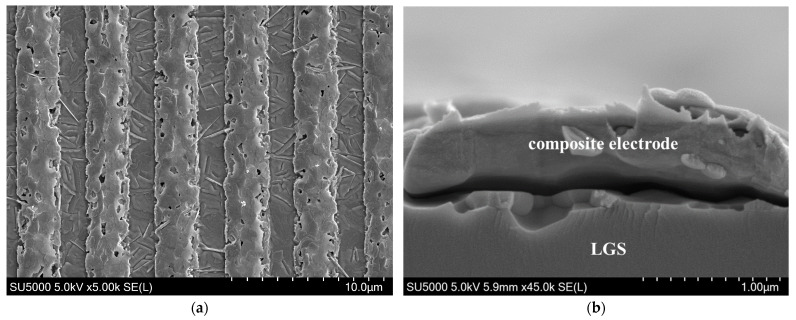
SEM images of Pt-10%Rh/Zr/Pt-10%Rh/Zr/Pt-10%Rh/Zr IDT after continuous wireless temperature measurement at 1200 °C for 3 h: (**a**) Surface morphology and (**b**) cross-sectional images.

The reasons for the failure of the LGS SAW sensor after continuous operation at 1200 °C for 2.18 h were also analyzed. As shown in Figure 13, even after the sudden change in the resonant frequency of the LGS SAW sensor, the reader was still able to read the resonant frequency of the LGS SAW sensor. This shows that at the time of the sudden change in the resonant frequency, the electrodes of the LGS SAW sensor did not lose conductivity, and the failure was not due to electrode metal agglomeration as reported in previous studies [11,24,25]. This inference is further confirmed by the SEM images of the LGS SAW sensor after continuous wireless temperature measurement at 1200 °C for 3 h in Figure 14. Compared to the surface morphology of the electrode in Figure 12c, the surface morphology of the electrode in Figure 14a remains relatively intact, with no fractures observed. Only partial agglomeration, a few voids, and some nano-whiskers originating from the multilayer thin-film composite electrodes are observed [33]. However, as seen in the cross-sectional image of the electrode in Figure 14b, after being exposed to 1200 °C for 3 h, the composite electrode has completely separated from the LGS substrate; at this moment, the reader can no longer receive effective echo signals from the LGS SAW temperature sensor. Therefore, it can be inferred that the failure of the LGS SAW temperature sensor after continuous operation at 1200 °C for 2.18 h (sudden change in resonant frequency) is due to partial separation between the multilayer composite electrode and the LGS substrate. This has weakened the mass loading effect of the composite electrode, leading to a sudden increase in the resonant frequency of the LGS SAW sensor [34]. Subsequently, the composite electrode further separates from the LGS substrate, causing the resonant frequency of the LGS SAW sensor to continue rising until the electromechanical coupling effect weakens to the point where effective echo peak signals can no longer be distinguished by the reader. Additionally, it can be observed that after 3 h of high-temperature treatment at 1200 °C, the composite electrode can no longer be distinguished as a multilayer structure. The zirconia pinning layer, formed by the oxidation of zirconium at high temperatures, has completely lost its pinning effect, and the multilayer metal electrodes have completely penetrated each other.

Since the LGS SAW sensor continued to send valid echo peak signals to the reader even after operating normally for over 2.18 h at 1200 °C, future work will focus on addressing the long-term adhesion issue between the composite electrode and the LGS substrate at this extreme temperature. This will aim to further extend the operational lifespan of the LGS SAW sensors.

Table 1 summarizes the current research progress in wireless temperature measurement using SAW sensors. It can be seen that the LGS SAW sensor developed in this study made significant advancements in temperature measurement range, achieving a measurement range of up to 1200 °C.

## 4. Conclusions

This study proposes a wireless high-temperature LGS SAW sensor using a Pt-10%Rh/Zr/Pt-10%Rh/Zr/Pt-10%Rh/Zr multilayer composite electrode structure, capable of wireless temperature measurement from room temperature to 1200 °C with an accuracy of 1.59% of the full scale. The sensor maintained its electrode integrity after full-range wireless temperature measurement. The composite electrode excites a quasi-shear wave mode with a Q-factor of 3526 at room temperature, providing an initial assurance for the strength of the wireless interrogation echo signal. The sensor demonstrated an operational lifespan of 2.18 h at 1200 °C, after which a sudden increase in resonant frequency rendered the original calibration model invalid. This increase was attributed to partial adhesion loss between the composite electrode and the LGS substrate after prolonged exposure, rather than electrode agglomeration or fracture. Future research will focus on ad-dressing the adhesion degradation issue between the composite electrode and the LGS substrate at 1200 °C, exploring alternative materials and fabrication methods to achieve a longer operational lifespan at this extreme temperature. These advancements will significantly benefit wireless temperature sensing in extreme environments, with applications in the aerospace, industry, and energy sectors.

## Figures and Tables

**Table 1:** Summary of state-of-the-art wireless temperature SAW sensors.

Electrode and Substrate Materials	Wireless Temperature Measurement Range	Refs.
Al/LiNbO_3_	25 ℃ to 200 ℃	[35]
Pt/Ti/LGS	25 ℃ to 700 ℃	[13]
Pt/Ti/LGS	Room temperature (RT) to 700 ℃	[14]
Al/Ti/Al/Ti/LiNbO_3_	30 ℃ to 200 ℃	[36]
Au/LiNbO_3_	25 ℃ to 95 ℃	[37]
Copper-doped Al/Quartz	RT to 480 ℃	[16]
Al/LiNbO_3_	−10 ℃ to 100 ℃	[15]
Al/Quartz	0 ℃ to 100 ℃	[17]
Pt-10%Rh/Zr/Pt-10%Rh/Zr/Pt-10%Rh/Zr/LGS	RT to 1200 ℃	This work

## Data Availability

The original contributions presented in the study are included in the article, further inquiries can be directed to the corresponding author.
